# Moral reasoning skills: what they are and how they can be furthered in health professions education

**DOI:** 10.1007/s11019-025-10289-0

**Published:** 2025-08-21

**Authors:** Annett Wienmeister

**Affiliations:** Institute for the History of Medicine and Ethics in Medicine, Charité - Universitätsmedizin Berlin, Berlin, Germany

**Keywords:** Moral reasoning, Ethics, Informal logic, Argument, Education

## Abstract

It is widely agreed that moral reasoning skills are an important aspect of ethical competency in the health professions and that students should acquire those skills. Nevertheless, ethics instructors might find it difficult to choose specific exercises and methods to further those skills because there is no shared understanding of what the term “moral reasoning skills” implies. As a result, there is a didactical gap between learning objective and methodology. In this paper, I demonstrate that and why the term “moral reasoning” is an underdetermined concept in the didactics literature of the health professions. With reference to the discipline of informal logic I will introduce a definition of the term and quality criteria for good moral reasoning that facilitate didactical interventions. I introduce three basic suggestions that instructors can follow if they want to further moral reasoning skills in students. I show how the three suggestions translate into specific learning objectives, which help instructors design exercises and choose appropriate methods for teaching and learning. Towards the end, I will discuss the critical factor of time in educational settings.

## Introduction

People working in health professions are faced with a multitude of ethical challenges in their daily work. As constantly developing technological inventions expand the realm of the feasible and as our societies become more and more culturally diverse, moral problems in the health care sector become increasingly complex. Scholars working in the field of ethics education point to the importance of teaching moral reasoning skills to future health professionals in order to help them deal with moral conflicts in their professional fields (Andersson et al. 2022; Buyx et al. 2008; Chachad et al. 2024; Foy et al. 2023; Giubilini et al. 2016; Langer et al. 2016). Being an important aspect of ethical competency, moral reasoning skills are considered to play a major role in critically analyzing ethical issues from different perspectives, bringing forth arguments and evaluating arguments of others (Giubilini et al. 2016, p. 132).

While it is widely agreed that health care professionals should acquire moral reasoning skills, ethics instructors may find it difficult to select appropriate methods for teaching and assessing moral reasoning (Tsai/Harasym 2010, p. 865). There is a didactical gap from the learning objective to specific exercises, techniques and methods that help students acquire those moral reasoning skills. One reason that explains the didactical gap is that theories of argumentation and moral reasoning have received only little attention in health professional (didactics) literature (Foy et al. 2023, p. 990; Labrie/Schulz 2014, p. 997; Rubinelli/Zanini 2012, p. 66). Furthermore, there is no shared understanding in the field what moral reasoning is in detail (Tsai/Harasym 2010, p. 864) and how it should be taught (Langer et al. 2016, p. 1). In this paper, I want to contribute to the question what moral reasoning skills are and how they can be furthered by reference to theories of argumentation theory and argumentation didactics.

This article begins with a few introductory remarks on ethical competency in the health professions and on moral reasoning as an important learning objective in educational programs. I will then demonstrate that and why the term “moral reasoning” is an undetermined concept in the didactics literature of health professions (part two). In chapter three of this paper, I will refer to informal logic, a subdiscipline within argumentation theory, in order to propose a definition of the term “moral argument”. In a next step, I will show how this definition allows us to derive two quality criteria for good moral reasoning. I will use these quality criteria in order to develop three recommendations that instructors can follow if they want to further moral reasoning skills in students. An example from a possible ethics discussion on active voluntary euthanasia in class will help illustrate the benefits of this approach. In the last chapter, I will make some practical remarks on specifying learning objectives and choosing appropriate methods as they derive from the definition of the term “moral argument” and from the three suggestions developed in this paper. I will also discuss the critical factor of time in educational settings.

## Moral reasoning skills as an element of teaching ethical competency in health professions: an underdetermined concept

Moral reasoning skills are a key feature of ethical competency in health professions. While it is difficult to present a clear-cut definition of the term “ethical competency”, several concept studies have found main elements that are repeatedly related to ethical competency in health professions. Amongst the elements are moral reasoning and judgment skills. According to a literature review that Kulju et al. conducted in 2015 in the context of health care, the term “ethical competency” can be “defined in terms of character strength, ethical awareness, moral judgement skills and willingness to do good” (Kulju et al. 2016, p. 406). This literature review summarizes moral judgement skills more closely as the ability “to consider critically and logically all values, principles, needs and beliefs; and making moral judgements consistently, from the alternatives involved in an ethically demanding situation.” (Kulju et al. 2016, p. 406). A second systematic review of literature about means to further nurses’ ethical competency by Poikkeus et al. identified different viewpoints from which ethical competency was addressed. Ethical reasoning, again, is one of them, next to “ethical decision-making, ethical sensitivity, ethical reflection, ethical knowledge, ethical behavior and ethical action.” (Poikkeus et al. 2014, p. 263; see also Lechasseur et al. 2018, p. 696 and Michl et al. 2022, p. 440).

Along with the importance of promoting ethical competency in health professions, the demand of teaching moral reasoning skills to students is widely acknowledged (Langer et al. 2016, p. 1). Buyx et al. reviewed literature discussing appropriate learning objectives in medical ethics education. They conclude that ethics teaching does and should include “(1) the teaching of moral reasoning skills, (2) the instruction about relevant ethical knowledge, as well as (3) the development of certain character traits.” (Buyx et al. 2008, p. 655) According to them, the development of moral reasoning skills in students relates to cognitive and discursive learning objectives. These objectives include the awareness of moral conflicts, the evaluation of a given situation and the involved arguments as well as moral judgement making. Amongst the objectives are also the ability to differentiate between different perspectives on a given situation, to identify the underlying principles and arguments and how they can be in conflict (Buyx et al. 2008, p. 657). Giubilini et al. evaluated medical ethics curricula in medical schools in Western English-speaking countries. Together with the ATEAM (the Association of Teachers of Ethics and Law in Australian and New Zealand Medical Schools), they highlight the need for students to learn critical reasoning skills, that is skills to “analyze ethical issues, construct arguments and counter arguments that are valid and sound, and examine and interpret the arguments of others” (Giubilini et al. 2016, p. 132). Michael L. Gross understands the teaching and learning of moral reasoning skills in medical ethics education as informed by moral philosophy. He defines moral reasoning as the “ability to not only articulate one’s viewpoint but to refute common objections and persuasively defend one’s point of view.” (Gross 2001, p. 389).

### A plurality of approaches to and methods for furthering moral reasoning skills

It is undisputed that moral reasoning skills are an element of ethical competency in health professions and that students should acquire those skills. At the same time, instructors of ethics in health professions might have a hard time translating learning objectives concerning moral reasoning skills into specific exercises as well as to choose appropriate methods for teaching and assessing them. Tsai and Harasym explain the challenge instructors face by the fact that moral reasoning in health professions is not very well understood (Tsai/Harasym 2010, p. 864). They point to the fact that published research on the topic comes from different disciplines with a variety of investigative approaches and that the contexts, in which moral reasoning figure, can vary widely. The difficulty for instructors described by Tsai and Harasym is reflected in the plurality of moral reasoning models they identify through a literature search in a variety of fields, such as nursing, medicine or psychology. They divide reasoning models into two groups: justification-based and task-based models. On the one hand, justification-based models subsume different approaches to moral reasoning such as Kohlbergs and Gilligans stage models of moral development, consequentialist and deontological models as well as reflective equilibrium approaches. On the other hand, task-based models refer to the steps of ethical decision making, such as Rests four-component model or different five, seven or ten step models (Tsai/Harasym 2010, p. 866). Looking at the variety of reasoning models that Tsai and Harasym describe, it becomes obvious that each of them addresses very different skill sets at different levels of abstraction. Consequentialist and deontological approaches operate at a micro level of reasoning, referring to consequences or duty and dignity when arguing for or against a specific viewpoint. Models of decision-making lead one through different stages of problem solving and Kohlbergs stage model refers to the moral development of a person. Given the plurality of skills, attitudes, and levels of abstraction that the different reasoning models address, it is understandable that instructors who do not have a background in theories of reasoning might have some challenges making their didactic choices.

In spite of the plurality of approaches to moral reasoning, there is a set of methods that is repeatedly suggested for fostering moral reasoning skills in students of health professions, such as (small size) group discussion, problem-based learning and case studies (Buyx et al. 2008, p. 657; Lin et al. 2010, p. 380; Giubilini et al. 2016, p. 137; Kuhn et al. 2021, p. 1). One can add simulation activities, narratives and storytelling (Andersson et al. 2022, p. 20) as well as role-plays to this list (Giubilini et al. 2016, p. 137; Chachad et al. 2024, p. 655). Furthermore, the use of decision-making models is often favored in the literature dedicated to teaching moral reasoning skills (Chowning et al. 2012, p. 1; Tsai/Harasym 2010, p. 868; Giubilini et al. 2016, p. 137; Friedrich et al. 2017, p. 2). It is intuitively clear that teaching these methods, formats and models can promote moral reasoning skills in one way or another, especially in comparison to lecture based ethics courses. However, the above-mentioned literature rarely engages in a more specific description of how exactly one can further morals reasoning skills by using these methods. For instance, they leave open what it means to justify one’s viewpoint or to counter objections. Without further clarification, reference to methods such as group discussion or decision-making models are good generic advice, but they leave many conceptual and subsequently didactical questions unanswered.

### Moral reasoning skills cannot be presupposed

One might assume that students at the university level have already acquired moral reasoning skills through prior education, i.e. at the high school level. Unfortunately, that assumption is not very well grounded. Research shows that students at the middle and high school level have problems to build arguments, to justify their claims and rebut contrary positions when discussing socio-scientific issues (Sadler 2004, p. 516; Zohar/Nemet 2002, p. 39). Secondary school teachers report that while biology text books often present a controversial issue and ask students to “justify their position”, many students lack an understanding of how a position or argument can be well-justified (Chowning et al. 2012, p. 7). In case this lack of understanding has not been addressed at the secondary school level or later at the University (i. e. in critical thinking courses), it most certainly will still obtain in students throughout their studies, leaving them “without skills to negotiate routine disagreements associated with contemporary health care contexts” (Foy et al. 2023, p. 990). In an evaluation of an extracurricular clinical ethics training for medical students at the University of Kiel, Germany, students who were to improve their moral judgement and ethical reflections skills using case studies and problem-based learning explicitly stated that they want more detailed information and support on argumentation methods (Kuhn et al. 2021, p. 4). A lack of competency regarding argumentation and moral reasoning might partly explain why students of health professions often experience ethics as a “fluffy” discipline (Giubilini et al. 2016, p. 135). It might also explain why they comment that “ethics discussions, while lots of fun, often lead nowhere. They want to see closure, just as they see when a challenging clinical case is presented. Instead they are often left with the impression that ethics can justify anything (or nothing).” (Gross 2001, p. 390).

One can summarize that it cannot be taken for granted that students of ethics classes in health professions already come equipped with moral reasoning skills. Furthermore, the term “moral reasoning” has multiple meanings in the literature dedicated to teaching moral reasoning skills in health professions. In addition, the literature does not engage in a more specific definition of what it means to further moral reasoning skills. The term is thus underdetermined for didactic purposes. Michael L. Gross comments that it is not clear “how to teach what has not yet been clearly defined” (Gross 2001, p. 387). He concludes that if the outcome of ethics education is that students are left with the impression that ethics can justify about anything, “then students might be better left to their own devices, guess work and intuition.“ (Gross 2001, p. 390).

## A little bit of informal logic: definition, quality criteria and three recommendations for the fostering of moral reasoning skills in health professional students

The fact that the term „moral reasoning” is an underdetermined concept in the didactics literature of the health professions is not reason enough to give up the corresponding learning objectives all together, as Gross provocatively suggests. While intuition and emotional sensibility most certainly are very important aspects of ethical judgement making and should have a place in the teaching of ethics (Macneill 2010; Lesser 2010; Sadler/Zeidler 2005), it is negligent to leave ethical judgement to “guess work”. Therefore, it is much more reasonable to clarify the term “moral reasoning” in a way that is of use to ethics instructors who want to address the learning objectives concerning moral reasoning mentioned above: analyze ethical issues in health care, make moral judgements, bring forth and evaluate arguments from a logical and critical perspective.

### Defining moral reasoning: a glance at the field of informal logic

When attempting to give a more precise definition of the term “moral reasoning” for the purpose of improving the teachability of corresponding skills, one can look to the discipline of informal logic. This field of study sets itself apart from formal logic, already by name. The latter uses formal/symbolic language to analyze thought and puts an emphasis on deductive inferences. Informal logic on the other hand aims at understanding and improving thinking and reasoning in “real life” and therefore in natural languages, such as English. Contexts, in which methods of informal logic are developed and applied are, i.e., “public discussion and debate, in education and intellectual exchange, in interpersonal relations, and in law, medicine and other professions.” (Groarke 2024) The term “informal logic” at times has been used interchangeably with “argumentation theory” (Walton 2009, p. 2). While both have common aims and subjects of research, one can distinguish the two disciplines from each other (Blair 2015, p. 39). Argumentation theory is the broader approach to reasoning and argumentation, taking into account insights from formal and informal logic, as well as from other fields of study, such as psychology, sociology, politics, rhetoric, communication theory and linguistics. Informal logic does not aim to analyze communicative or pragmatic aspects of arguments and reasoning. It focusses on the interpretative tasks of recognition and reconstruction of arguments and on their assessment in epistemic and logical terms. As will become clear below, important aspects of argument assessment in this field amount to premise acceptability and inferential strength. Informal logic is thus the more narrow field of study and can be considered a part of argumentation theory, the latter of which comprises not only logical and epistemological, but also rhetorical, practical, aesthetic, or dialectical approaches to argumentation and reasoning.

In what follows, I will refer mainly to insights in and guidelines for argumentation and reasoning that come from the field of informal logic. This is not meant to discredit other approaches to reasoning, such as rhetorical or pragmatic accounts, which I believe to be as important to the teaching of medical ethics as the approach from informal logic. There is, however, a specific merit of the contribution of informal logic to the task of this paper: informal logic provides us with clearly defined criteria for analyzing and evaluating arguments that can be translated fairly easily into teaching methods. Furthermore, while there is already an established field of research in health care that draws on the broader field of argumentation theory, i.e. on the pragma-dialectical approach for the context of medical consultation (Labrie/Schulz 2014; Rubinelli/Zanini 2012) or for the context of health policy decision making (Rubinelli/Groote 2017), methods and guidelines from informal reasoning have not been widely acknowledged in the health professional (educational) literature. This paper therefore wants to make a contribution to the field.

When it is the aim to implement knowledge and methods from informal logic into the field of health professional education, it is reasonable to relate to a discipline that puts an emphasis on teaching informal logic to students, namely philosophy. And indeed, the following definitions, quality criteria and recommendations will be familiar to students of philosophy and ethics, as well as to students of critical thinking classes and alike. But one has to keep in mind the following major difference between teaching informal logic to students of philosophy and to students of the health professions: When students of philosophy study informal logic in class, they often have lectures and tutorials over several semesters. Due to time constraints and the purpose of ethics education in the health professions, it is unsuitable and unrealistic to adopt the whole agenda of philosophical reasoning courses and transfer it to ethics classes in this field. In what follows, I make realistic suggestions of how to teach fundamental moral reasoning skills that are of relevance for the health professions, without presupposing prior knowledge (i.e. in formal logics or critical reasoning), and which can be implemented in the short amount of time usually available for ethics courses in the curriculum.

### What is an argument? The abstract propositional sense

The term ‘argument’ can have many meanings in the broad field of argumentation theory, depending on whether one refers to its logical, rhetorical, pragmatic or dialectical dimension. In the field of informal logic, a rather narrow definition suggests itself. Walton introduces what he calls the “minimal inferential definition”, according to which “an argument is a set of statements (propositions), made up of three parts, a conclusion, a set of premises, and an inference from the premises to the conclusion.” (Walton 2009, p. 2) The minimal differential definition leaves it open whether an argument is actually becoming part of an actual speech act, through which a person articulates the argument in speech or in written form. Accordingly, the entry on ‘argument and argumentation’ in the *Stanford Encyclopedia of Philosophy* distinguishes a speech act sense from an abstract propositional sense of ‘argument’:

An argument can be defined as a complex symbolic structure where some parts, known as the premises, offer support to another part, the conclusion. Alternatively, an argument can be viewed as a complex speech act consisting of one or more acts of premising (which assert propositions in favor of the conclusion), an act of concluding, and a stated or implicit marker (“hence”, “therefore) that indicates that the conclusion follows from the premises (Dutilh Novaes 2022, pp. 2–3).

Next to the distinction of the abstract propositional sense of the term ‘argument’ and the speech act sense, one can also differentiate the term ‘argument’ as it is used in the sense of ‘argumentat*ion*’. ‘Argumentation’ refers to the “interpersonal activity in which reasons for beliefs, opinions, and policy proposals are considered, discussed, and exchanged and, in the good case, defended and criticizes by way of arguments” (Siegel 2023, p. 471). Argumentation therefore does involve arguments in the abstract propositional sense and in the speech act sense, but it “picks out the communicative, dialectical/dialogical social practices and activities.” (Siegel 2023, p. 473) Finally, a fourth meaning of the term ‘argument’ should be mentioned, which is used quite often in every day English: the meaning of ‘argument’ as in ‘disagreement’, ‘quarrel’, ‘controversy’, and ‘fight’. While a disagreement at best does involve arguments in the propositional sense and in the speech act sense by means of argumentation, it might also be the case that this kind of communicative activity does not involve arguments in the three above senses at all.

Clarifying the different meanings of the term ‘argument’ (the propositional sense, the speech act sense, the sense of argumentation, the sense of quarrel) might seem a bit specific for the purpose of furthering moral reasoning skills in health professions education. Nevertheless, this distinction helps us identify quality criteria for good moral reasoning that allow us to specify what we expect our students to learn—namely to analyze and evaluate arguments critically. Before identifying quality criteria for good moral reasoning, I would like to introduce a definition of the term “moral reasoning” in the sense of ‘moral argument’.

### What is a *moral* argument?

Let us remember the propositional sense of argument. It will be the base of our considerations in this paper: an argument is a complex symbolic structure where some parts, known as the premises, offer support to another part, the conclusion. To give you an example: “All bioethicists are human (premise). Tatyana is a bioethicist (premise). Therefore, Tatyana is human (conclusion).” Another example might be “When a patient does not consent to a certain treatment, then one should not give the treatment to the patient (premise 1). Tatyana does not consent to a certain treatment (premise 2). Therefore, one should not give the treatment to Tatyana (conclusion).” The second argument is a moral argument, because it contains statements about what should or should not be done (premise 1, conclusion). Moral arguments in general are “arguments about what is right or wrong, what should or should not be done, or what is moral or immoral.” (Feldman 2014, p. 368) Accordingly, moral propositions that make up a moral argument say „that something is moral or immoral, right or wrong, or should or should not be done.” (Feldman 2014, p. 371). Although there are differences in meaning between ‘being moral’, ‘being right’ and ‘should be done’, they all can be considered to be moral statements in a normative sense, because they imply some sort of moral norm. Other kinds of normative statements refer to other norms, such as “This painting is beautiful” (aesthetic norm) or claims referring to functions and/or means-end relations, such as “In order to lower the risk of heart attack, you should lower your blood pressure.” Normative statements have in common that they imply some norm that can be realized in a given instance, but it does not necessarily have to be or will be. That sets normative statements—moral or functional–apart from descriptive or factual statements. The latter say what is the case, not what should be.

In philosophy, there is a lot of research on what makes a proposition normative as opposed to descriptive and a normative proposition a moral one as compared to, i.e., an aesthetic one. For the purpose of this paper, it is sufficient to refer to the rather common and intuitive understanding of the term “moral” described above. What is more, the means and quality criteria to analyze and assess moral arguments that I will introduce below apply to all normative and descriptive arguments alike and are therefore general means of argument evaluation.

### Quality criteria for arguments and three recommendations for the fostering of moral reasoning skills

It is widely agreed in the broad field of argumentation theory that there is not just one single model for the interpretation and evaluation of arguments. Arguments “can be evaluated in terms of epistemic strength, rhetorical or persuasive force and effect, ability to bring about consensus, aesthetic properties, or along yet other dimensions”, such as “dialectical propriety, arguers’ virtues, arguers’ goals/purposes.” (Siegel 2023, pp. 488 and 511) As this paper emphasizes the abstract propositional sense of ‘argument’, it is not so much aesthetic or dialectical and communicative criteria that come into focus, but epistemic and logical ones. As Tracy Bowell and Justine Kingsbury put it in a nutshell:

A good argument is an argument that provides, via its premises, sufficient justification for believing its conclusion to be true or highly probable, or for accepting that the course of action it advises is one that certainly or highly probably should be taken. This account of good argument has both logical and epistemic elements. (2013, p. 23)

What makes a good argument from the epistemic and logical approach is its ability to secure the epistemic status of its conclusion in the sense that it is rational to believe what is stated in the conclusion, precisely because what is stated in the premises is considered to be true, highly probable, right or should be done. Within this rather narrow propositional sense of ‘argument’, there are two ways an argument can fail: either the premises are not worthy of our acceptance or the proposed premises are not properly connected to the conclusion (Feldman 2014, p. 191). When we reject premises, i.e., by denying their being true, they lose their legitimizing force for the conclusion. When we analyze the inference relation from the premises to the conclusion, we ask whether the conclusion follows from the premises, independent of our acceptance of the premises. The main two quality criteria therefore are “premise acceptability” and “inferential strength” (Blair 2015, p. 32). How exactly the two quality criteria figure in argument interpretation and evaluation becomes clearer when we look at an example of a possible discussion that could take place in an ethics class.

### An example: a possible discussion on voluntary active euthanasia in the classroom

Consider a debate on voluntary active euthanasia. Voluntary active euthanasia means that an individual wants to be treated with a lethal medication, given by another person, in order to end his or her life. One can imagine that after a short introduction into medical and legal issues (or a research phase where students gather information of that kind themselves), the instructor asks of the students their opinion whether voluntary euthanasia is ethically permissible or not. In case the instructor merely asks of the students to justify their position, skills of moral reasoning are rather presupposed than promoted in class. Unfortunately, as we have seen, one cannot take for granted that students already come equipped with moral reasoning skills, even at the University level. Therefore, a didactical framing of the debate is helpful, a framing that refers to standards and quality criteria of argument interpretation and evaluation from within informal logic.

Let’s have a look at two possible contributions to the discussion (inspired by Debbie Newman and Ben Woolgar 2014, p. 119). They could be oral contributions of students in class or written contributions in newspaper articles or elsewhere. Ideally, one can work with written statements, because one has more time and opportunity to focus on the argumentative structures.

Contribution I:

How long do we want people to suffer? And who wouldn’t want to be able to request a ‘mercy killing’ to end their suffering? I mean, people, in the later stages of severe illnesses, face more and more physical degeneration and acute suffering. They should be allowed to die with dignity with the help of, for example, a lethal injection or an overdose of morphine, given by a doctor.

Contribution II:

What a burden! This amounts to compromising the medical professional oath. And really, who wants to be put under the great moral pressure of deciding whether to grant a particular patient voluntary euthanasia or not? With the highly effective painkillers now available, there is never any need even for the terminally ill to suffer great pain. Use of painkillers, not euthanasia, is the answer to painful terminal illness.

It is intuitively clear that contributions I and II are arguments for and against voluntary euthanasia. One possible next step in class could be to gather more arguments for and against voluntary euthanasia and write them down in a list of Pros and Cons. Such a list might give students a quick overview of possible different takes on a given subject, but if one stops here, a lot of potential to further moral reasoning skills is left unused. What is more, if one does not evaluate the arguments more closely, students might be left clueless as what to think because there are arguments for both sides. Does the side with the most arguments win? That is not naturally the case and a more thorough analysis of the arguments is needed if one wants to equip students with moral reasoning skills.

At this point, I would like to develop the three recommendations for teaching moral reasoning skills along the two quality criteria for moral arguments: “inferential strength” and “premise acceptability”. I would like to begin with the criterion of “inferential strength”. When one analyzes the inferential strength of an argument, one asks whether the conclusion follows from the premises.^1^ Imagine an instructor asks of students to answer this question with relation to contribution I. It is highly probable that many of them will have trouble to give a qualified answer. That is of no surprise because at first sight it might not be clear which of the statements of contribution I serve as a conclusion and which as premises. In order to be able to assess the inferential strength of an argument, the first recommendation therefore is:

**Recommendation I: secure the argument by stating the conclusion and premise(s)**

It can be tricky to identify the conclusion and premises of an argument. Whether a statement is a conclusion or a premise is not inherent in the statement itself, but it results from its relation to the other statements of the argument. Therefore, a statement that is the conclusion of one argument can figure as a premise in another argument, and it often does. So, one really has to analyze the statements of an argument in their relation to the other statements in order to find out which is which. Sometimes, one will find logical indicators: Words that indicate premises are, i.e., “because”, “since”, or “for”. Words that come before the conclusion might be “therefore”, “hence”, “thus” or “as a result”. Unfortunately, in our examples, no logical indicators can be found.

It is often helpful to start the analysis by looking for the conclusion. The first contribution begins with two leading questions: “How long do we want people to suffer? And who wouldn’t want to be able to request a ‘mercy killing’ to end their suffering?” As leading questions, they at best hint at possible claims, but they don’t state clearly that something is or should be the case. A conclusion needs to be formulated as a complete descriptive or normative statement. Questions, as well as mere exclamations, cannot serve as conclusions nor premises—they would have to be reformulated as full statements. Let’s look at the other sentences of contribution I. As the discussion in class circles around an ethical evaluation of voluntary active euthanasia, one would expect a moral statement as the conclusion. And there is one: “They should be allowed to die with dignity with the help of, for example, a lethal injection or an overdose of morphine, given by a doctor.” After one has identified the conclusion (or even added one in case it was implicit), one can look for the premises. In the first contribution, there is only one statement left that can serve as premise and it makes a factual claim about physical degeneration and suffering in late stages of illness. We have now secured the argument by stating explicitly which statement is the conclusion and which statement serves as a premise.

At this stage of the argument reconstruction, students of a philosophy class are often asked to reconstruct the argument in standardized form. If time allows it, this is also a valuable exercise in ethics classes of the health professions and it will become clear below why. One way to reconstruct an argument in standard form is to list the premises in full sentences, mark them as such and write the conclusion underneath. For our example:

P: People, in the later stages of severe illnesses, face more and more physical degeneration and acute suffering.

C: They should be allowed to die with dignity with the help of, for example, a lethal injection or an overdose of morphine, given by a doctor.

While writing down an argument in standard form is certainly not necessary in each debate and might take extra time, spelling out premises and the conclusion this way adds clarity to an argument. For if you look at the standardized argument now, it becomes obvious what might not have been obvious in the first place: The premise alone does not secure the conclusion. It remains open why a person who believes in the premise should also accept the conclusion. Obviously, other premises are missing and the inferential strength of the argument is limited. Therefore, a second recommendation is:

**Recommendation II: complete the argument more or less**

Oftentimes, there are several premises missing to an argument. Not all of them necessarily need to be spelled out, especially the ones that are obvious and undisputed by all parties. However, it is advisable to spell out contested premises. When we look at contribution I, one premise that is missing is about the relation of dying in dignity and receiving voluntary active euthanasia. How one spells out this relation in detail might differ, one version could be: “To die in dignity means to undergo voluntary active euthanasia”, another one could be “One way to die in dignity is voluntary euthanasia”. Both statements say different things and one can see how they change the meaning of the argument. Argument reconstruction is therefore often an interpretative act. I will choose the second claim about the relation of dignity and voluntary active euthanasia at this point because it is the less contested one. Let’s have a look at the standardized form of the argument again:

P1: People, in the later stages of severe illnesses, face more and more physical degeneration and acute suffering.

P2: One way to die in dignity is voluntary euthanasia.

C: They should be allowed to die with dignity with the help of, for example, a lethal injection or an overdose of morphine, given by a doctor.

Even after adding premise 2, the argument is not yet complete in a way that would allow us to say that the conclusion follows from the premises. Another premise about the relation of the suffering of people and dying in dignity is missing. What is more, premises 1 and 2 are descriptive statements, while the conclusion makes a normative moral claim. This amounts to the is-ought fallacy, in which one concludes a normative moral statement on the grounds of merely descriptive premises. Students of the health professions should be familiar with the is-ought fallacy in order to detect it and reply to it in every day reasoning in professional contexts. An appropriate way to deal with this fallacy is to apply the “principle of charity”. This principle states that an argument should be reconstructed in the strongest ways possible before discussing it in order to facilitate mutual understanding (Davidson 1973–1974, p. 19). It asks us to reconstruct premises and conclusions in a rational and coherent way. In order to do so, on should add relevant premises and ignore irrelevant ones. The goal here is to protect the argument from being an is-ought fallacy. In an oral discussion, this amounts to inquiring of someone to lay open the missing normative premise. In our case, it amounts to adding it to the best of our knowledge. Here is a suggestion: “People in later stages of their illness, who face more physical degeneration and acute suffering, should be allowed to die in dignity.” At this point, we now have a more or less complete argument. One could furthermore specify the relation of voluntary active euthanasia and lethal injections of overdoses of morphine, but I will not go into that much detail for the purpose of this paper:

P1: People, in the later stages of severe illnesses, face more and more physical degeneration and acute suffering.

P2: People in later stages of severe illness, who face more physical degeneration and acute suffering, should be allowed to die in dignity.

P3: One way to die in dignity is voluntary euthanasia.

C: They should be allowed to die with dignity with the help of, for example, a lethal injection or an overdose of morphine, given by a doctor.

While the second recommendation on completion holds for any argument, it can be of particular importance in the case of moral reasoning. Even though we assume that a group of people agrees on a fair amount of fundamental moral norms and values, this is by means not true for every moral statement. Often, it is exactly the normative premise of a moral argument that is subject to debate. It is therefore recommended to make missing normative premises explicit in order to verify whether one should discuss them in more detail or not. The second recommendation therefore brings us directly to the last and third recommendation, which refers to quality criterion number two “premise acceptability”:

**Recommendation III: locate and formulate criticism of premises precisely**

When we go back to contribution I, the normative premise 2 is probably not controversial. Many people would agree that people in later stages of severe illness should be allowed to die in dignity. Premise 3 is more likely to cause disagreement: Is it acceptable to believe that one way to die in dignity is voluntary euthanasia? Does anything speak against the idea that to undergo voluntary euthanasia is a way to die in dignity? A critical analysis of premise 3 will bring a shift in focus towards the question what it means to die in dignity. This shift might lead us away from our original argument to some degree, but it helps us locate and formulate criticism precisely. It points to topics underlying a debate that need to be discussed more thoroughly.

Another premise of contribution I that might cause questions and debate is the first premise “People, in the later stages of severe illnesses, face more and more physical degeneration and acute suffering.” In this statement, the terms “acute suffering” and “physical degeneration” are not precise and therefore need clarification. Conceptual clarity is important because the two terms figure in a premise that serves as a reason to justify voluntary active euthanasia, a practice in health care that is highly contested. By going back to contribution II, one can illustrate the disagreement well:

Contribution II:

What a burden! This amounts to compromising the medical professional oath. And really, who wants to be put under the great moral pressure of deciding whether to grant a particular patient voluntary euthanasia or not? *With the highly effective painkillers now available, there is never any need even for the terminally ill to suffer great pain.* Use of painkillers, not euthanasia, is the answer to painful terminal illness.

As we can see now, only the highlighted statement in italic is a direct answer to premise 1 of contribution I and it challenges it in parts. It states that because of effective painkillers, terminally ill people never have any need to suffer great pain. One benefit of recommendation three, which encourages us to locate criticism precisely, is that one can pin down and formulate disagreement as well as agreement precisely. In our case, this amounts to discussing further what counts as great pain and what is needed in order to determine whether someone suffers it or not.

Contribution II contains more statements. A close argument reconstruction (in standard form) helps recognize that reference to the professional oath and to moral pressure for physicians are separate arguments for the conclusion that voluntary euthanasia is problematic and they should be analyzed separately. If time permits, one can analyze in more detail the relation of different arguments, i.e. by using argument diagrams and maps (Reed et al. 2007). I will not go into details of this method here, but I would like to point out that some of the competencies that underlie the use of argument maps are addressed by the three recommendations above.

The criterion of premise acceptability addresses the question whether premises are true, reasonably probable or at least acceptable (Blair 2015, p. 32f.) However, premises can also be problematic because they presume the truth of the conclusion. This fallacy of circularity (begging the question, petitio principii) is problematic because what is to be proved (conclusion) is already assumed in the premise. As a result, the justificatory function of the premise is cancelled. Teaching the fallacy of circularity is a great opportunity to illustrate to students the justificatory function that premises have and the need for reasons that lie beyond the conclusion (see Cummings 2020 for more fallacies that are relevant in the health professions).

In this chapter, I have developed three recommendations for teaching moral reasoning skills along the two quality criteria for good (moral) arguments from the field of informal logic: “inferential strength” and “premise acceptability”. The three recommendations are:

Recommendation I: secure the argument by stating the conclusion and premise(s)

Recommendation II: complete the argument more or less

Recommendation III: locate and formulate criticism of premises precisely

In what follows, I will say a few words on how to translate the three recommendations into specific learning objectives, which help ethics instructors design exercises and choose appropriate methods for teaching and learning.

## Practical remarks on learning objectives and methods

The three recommendations above address very basic reasoning skills that students of the health professions should acquire when they are to reach the generally accepted learning objectives described at the beginning of the paper: analyze ethical issues in health care and bring forth and evaluate arguments from a critical perspective. Having in mind how the three recommendations have been developed, it is easy to specify very concrete learning objectives that are precise enough to facilitate didactic decisions.

### Specific learning objectives for furthering moral reasoning skills

Students should

- know and be able to apply the terms (moral) argument, premise, and conclusion
- know the difference between normative and descriptive statements and arguments
- be able to reconstruct an argument more or less (one’s own and of others)
- know and be able to apply criteria for good arguments: inferential strength (completeness) and premise acceptability
- be able to recognize, avoid and respond to fallacies: is-ought-fallacy, fallacy of circularity
- know and act according to the principle of charity

How exactly specific methods, exercises and forms of assessment can be aligned with these concrete learning objectives is worth an own paper. At this point, I would like to make some remarks that exemplify how the learning objectives can be addressed in class.

The introduction of definitions and concepts should always be intertwined with statements, arguments and discussions that students can relate to. Because bringing forth reasons is an activity humans constantly do, it is rather easy to connect to personal experiences and prior understanding of students. Topics at best come from within the professional field, but they can as well come from other interesting areas of everyday life.

When attempting to introduce the professional term “argument”, it is advisable to begin with introducing the terms “premise” and “conclusion” first. Exercises that further the ability to recognize and develop premises and conclusions can have different levels of difficulty. One can provide students with premise-conclusion-pairs without displaying which is which and let them decide. One can let them find premises and conclusions in a given text, under the use of logical indicators, such as “because” and “therefore”, or without. The competency to reconstruct an argument can also be practiced in different degrees of difficulty. One can start by displaying some of the premises of a complete argument and let students fill in the missing ones. Or one can lay open all the premises of a complete argument and let students fill in the missing conclusion. Later, one can present parts of a discussion, i.e. out of a newspaper article, and have students find and reconstruct complete and incomplete arguments. In case students reconstruct incomplete arguments, which most arguments will be in a given debate, one can then address the criterion of completeness and the importance to add premises. Introducing the is-ought-fallacy connects very well to this step. Next to interpreting arguments of others, students can also be encouraged to bring forth their own (complete) arguments. When it comes to the critical evaluation of arguments, students should be able to apply the two criteria for good arguments. This means that they should be able to decide whether an argument is missing important or controversial premises and which of the premises are in need of critical discussion. Again, they can do this with the argument of others or with their own.

When designing exercises, instructors should frame questions such that students know which concepts and criteria they should use to answer them. For instance, instead of merely asking which arguments one can find in a given text, one can ask of the students to find and secure the arguments by writing down premises and conclusions. Instead of merely requesting of the students to discuss arguments critically, one could formulate exercises such that students have to locate criticism precisely (Which premise is critical? Are important premises missing? Is the conclusion made explicit?) It is generally advisable to design exercises that build on each other, starting with easy ones, continuing with more challenging ones.

The learning objectives and recommendations I have developed in this paper are very basic. They determine a minimal set of skills that I believe students of health professions should have if they are to acquire moral reasoning skills. It is of course possible to aspire more complex learning objectives (see Burkard et al. 2021 for a systematic, spiral curricular framework for teaching and learning reasonings skills). But again, as ethics teaching in the health professions is faced with considerable time constraints, one has to be realistic as to what can be achieved in the given time.

I would like to finish with a few words on the issue of time. Engaging in deep discussions as well as the teaching and learning of reasoning skills are time-consuming activities (Chachad et al. 2024, p. 658; Rubinelli/Zanini 2012, p. 77; Andersson et al. 2022, p. 19). If students are to acquire these skills, repeated educational interventions are necessary. The recommendations for furthering moral reasoning skills that I make in this paper can therefore be best integrated into courses that take place more than once, i.e. in weekly seminars during a term or semester. Due to the fact that the three recommendations address a very basic skill set, it is very likely that one can integrate an exercise or two in a course and raise the level of difficulty step by step. I am aware that there are ethics courses that take place only once for a limited duration of time (i.e. 90 min). It is not entirely impossible to address some of the specific learning objectives in these “one-time-only-formats”. Instructors could, i.e., present their students a fully reconstructed argument from a text and ask which of the premises they would like to discuss. But it is rather unlikely that students will have the opportunity to sharpen their skills in this short amount of time.

Even under the restrictions of limited time for ethics education, there are advantages that come with the three recommendations developed above. As they address a very basic set of moral reasoning skills that can be taught in various “little steps”, one can integrate exercises to further those skills in different courses along the curriculum. Furthermore, the fact that one can teach those skills almost irrespective of the given topic of a course facilitates repeated didactic interventions.

## Conclusion

In this paper, I argue that the term “moral reasoning” is an underdetermined concept in the didactics literature of the health professions. While it is widely accepted that future staff of the health professions should acquire moral reasoning skills in order to face ethical challenges in their professions, ethics instructors might have difficulties to determine what exactly moral reasoning skills are and which exercises and methods are useful to further them in students. With this paper, I contribute to closing this didactical gap by reference to the discipline of informal logic. I propose a definition of the term “moral argument” along with quality criteria for good moral reasoning that serve as the basis for three suggestions that instructors can follow if they want to further moral reasoning skills. I specify concrete learning objectives that are precise enough to help with choosing appropriate methods and exercises. As the three suggestions address a very basic skill set that can be taught in various little steps, it is possible to implement corresponding didactic interventions in ethics courses even under the restriction of limited time.

## Footnotes

1 In informal logic, one distinguishes deductively valid arguments, in which the truth of the premises secures the truth of the conclusion, from non-deductively (inductively) strong arguments, in which the truth of the premises makes it probable that the conclusion is true. Though this is an important distinction to know for philosophy students, I believe that students of the health professions do not necessarily need to be familiar with it. The following two recommendations serve the quality criterion “inferential strength” at a more basic level. They aim at making an argument complete, thereby pointing to implicit premises, which can then be subject to debate.
